# Dimethyl 2-amino­biphenyl-4,4′-di­carboxyl­ate

**DOI:** 10.1107/S1600536813010775

**Published:** 2013-04-27

**Authors:** Ryan L. Lehane, James A. Golen, Arnold L. Rheingold, David R. Manke

**Affiliations:** a Department of Chemistry and Biochemistry, University of Massachusetts Dartmouth, 285 Old Westport Road, North Dartmouth, MA 02747, USA; b Department of Chemistry, University of California, San Diego, 9500 Gilman Drive, La Jolla, CA 92093, USA

## Abstract

The title compound, C_16_H_15_NO_4_, exhibits two near-planar aromatic ester groups with a maximum aryl–ester torsion angle of 1.9 (2)°. The dihedral angle between the benzene rings is 44.7 (1)°. In the crystal, N—H⋯O hydrogen bonding is observed along with C—H⋯O contacts, forming chanins along [101]. No π–π inter­actions were noted between the benzene rings.

## Related literature

For the synthesis of the title compound, see: Olkhovik *et al.* (2008 ▶). For the crystal structures of the parent dimethyl-4,4′-di­carboxyl­ate and its structurally characterized amino derivatives, see: Ritzerfeld *et al.* (2009 ▶); Nyburg *et al.* (1988 ▶). For metal-organic framework structures with this and related linkers, see: Deshpande *et al.* (2010 ▶); Lun *et al.* (2011 ▶); Gupta *et al.* (2012 ▶); Sudik *et al.* (2005 ▶).
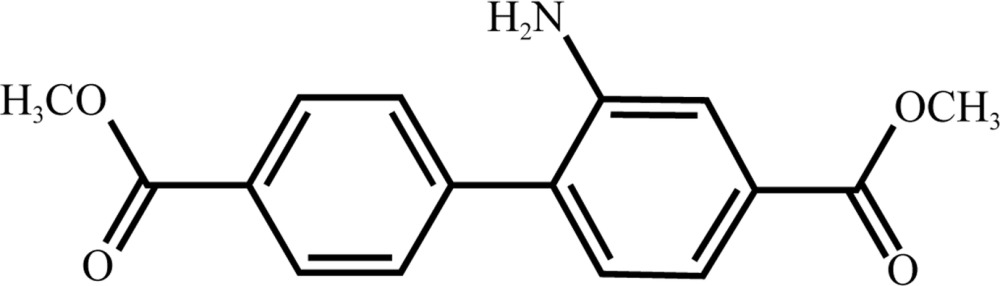


## Experimental

### 

#### Crystal data

C_16_H_15_NO_4_
*M* *_r_* = 285.29
Monoclinic, 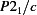

*a* = 12.955 (3) Å
*b* = 7.3460 (16) Å
*c* = 14.422 (3) Å
β = 103.263 (10)°
*V* = 1336.0 (5) Å^3^
*Z* = 4
Mo *K*α radiation
μ = 0.10 mm^−1^
*T* = 90 K
0.28 × 0.12 × 0.06 mm

#### Data collection

Bruker APEXII CCD diffractometer
Absorption correction: multi-scan (*SADABS*; Bruker, 2005 ▶) *T*_min_ = 0.972, *T*_max_ = 0.994
9113 measured reflections
2463 independent reflections
1785 reflections with *I* > 2σ(*I*)
*R*_int_ = 0.036

#### Refinement

*R*[*F* ^2^ > 2σ(*F* ^2^)] = 0.045
*wR*(*F* ^2^) = 0.124
*S* = 1.01
2463 reflections
198 parameters
2 restraints
H atoms treated by a mixture of independent and constrained refinement
Δρ_max_ = 0.27 e Å^−3^
Δρ_min_ = −0.35 e Å^−3^

### 

Data collection: *APEX2* (Bruker, 2005 ▶); cell refinement: *SAINT* (Bruker, 2005 ▶); data reduction: *SAINT*; program(s) used to solve structure: *SHELXS97* (Sheldrick, 2008 ▶); program(s) used to refine structure: *SHELXL97* (Sheldrick, 2008 ▶); molecular graphics: *SHELXTL* (Sheldrick, 2008 ▶); software used to prepare material for publication: *SHELXTL*.

## Supplementary Material

Crystal structure: contains datablock(s) I, global. DOI: 10.1107/S1600536813010775/ff2103sup1.cif

Structure factors: contains datablock(s) I. DOI: 10.1107/S1600536813010775/ff2103Isup2.hkl

Supplementary material file. DOI: 10.1107/S1600536813010775/ff2103Isup3.cml

Additional supplementary materials:  crystallographic information; 3D view; checkCIF report

## Figures and Tables

**Table 1:** Hydrogen-bond geometry (Å, °)

*D*—H⋯*A*	*D*—H	H⋯*A*	*D*⋯*A*	*D*—H⋯*A*
N1—H1*NB*⋯O2^i^	0.88 (1)	2.54 (2)	3.304 (3)	146 (2)
N1—H1*NA*⋯O4^ii^	0.88 (1)	2.33 (1)	3.147 (2)	155 (2)
C4—H4*A*⋯O2^i^	0.95	2.44	3.301 (3)	150

Symmetry codes: (i) 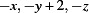; (ii) 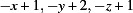.

**Fig. 1.**
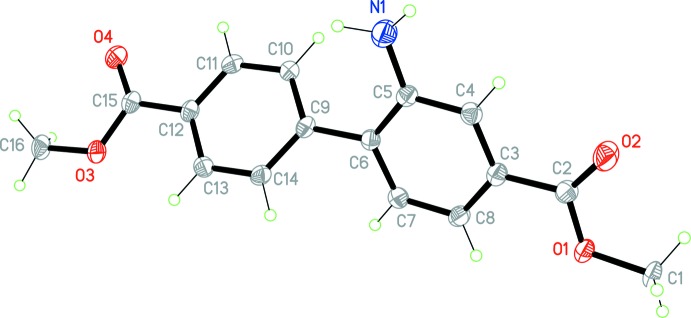
Molecular structure of the title compound, showing the atom labelling scheme. Displacement ellipsoids are drawn at the 50% probability level. H atoms are presented as spheres of arbitrary radius.

**Fig. 2.**
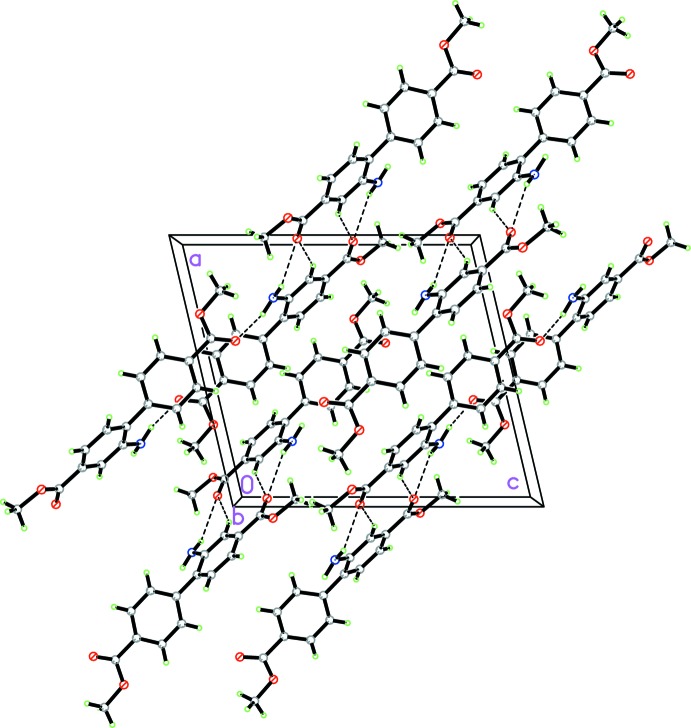
Molecular packing of the title compound with hydrogen bonding shown as dashed lines.

## supplementary crystallographic information

### Comment

Biphenyl-4,4'-dicarboxylate and its derivatives are widely used in metal-organic (MOFs) frameworks as linkers (Sudik *et al.* 2005). One of the many advantages of MOFs is the ability to incorporate different functional groups within their pores. An area of interest is the inclusion of open Lewis base sites on the interior surfaces of MOFs. As a part of our efforts in this field, we prepared the previously reported dimethyl-2-aminobiphenyl-4,4'-dicarboxylate (Olkhovik *et al.* 2008) and report its structure herein.

The molecular structure of the title compound is shown in Figure 1. The two phenyl rings demonstrate a dihedral angle of 135.3°. In the crystal, intermolecular hydrogen bonding is observed between N1–H1NA···O4 and N1H1NB···O2. There is also a close C–H···O contact along C4H4A···O2. No π-π interactions were noted between the phenyl rings. The packing for the title compound indicating hydrogen bonding is shown in Figure 2.

### Experimental

The compound was prepared by literature procedure (Olkhovik *et al.* 2008). Crystals suitable for single-crystal X-ray analysis were grown by slow evaporation of an ethanol solution.

### Refinement

All non-hydrogen atoms were refined anisotropically (*SHELXL*) by full matrix least squares on F^2^. Hydrogen atoms H1NA and H1NB were found from a Fourier difference map and were refined with fixed distance of 0.87 (005) Å and isotropic displacement parameter of 1.20 times *U*_eq_ of parent N atom. All other hydrogen atoms were placed in calculated positions and then refined with riding model with C—H lengths of 0.95 Å for (CH) and 0.98 Å for (CH_3_) and with isotropic displacement parameters set to 1.20 and 1.50 times *U*_eq_ of the parent C atom.

### Crystal data

**Table:**

C_16_H_15_NO_4_	*F*(000) = 600
*M**_r_* = 285.29	*D*_x_ = 1.418 Mg m^−^^3^
Monoclinic, *P*2_1_/*c*	Mo *K*α radiation, λ = 0.71073 Å
Hall symbol: -P 2ybc	Cell parameters from 2401 reflections
*a* = 12.955 (3) Å	θ = 2.9–24.9°
*b* = 7.3460 (16) Å	µ = 0.10 mm^−^^1^
*c* = 14.422 (3) Å	*T* = 90 K
β = 103.263 (10)°	Plate, colourless
*V* = 1336.0 (5) Å^3^	0.28 × 0.12 × 0.06 mm
*Z* = 4	

### Data collection

**Table:**

Bruker APEXII CCD diffractometer	2463 independent reflections
Radiation source: fine-focus sealed tube	1785 reflections with *I* > 2σ(*I*)
Graphite monochromator	*R*_int_ = 0.036
φ and ω scans	θ_max_ = 25.5°, θ_min_ = 3.0°
Absorption correction: multi-scan (*SADABS*; Bruker, 2005)	*h* = −15→15
*T*_min_ = 0.972, *T*_max_ = 0.994	*k* = −8→8
9113 measured reflections	*l* = −17→17

### Refinement

**Table:**

Refinement on *F*^2^	Primary atom site location: structure-invariant direct methods
Least-squares matrix: full	Secondary atom site location: difference Fourier map
*R*[*F*^2^ > 2σ(*F*^2^)] = 0.045	Hydrogen site location: inferred from neighbouring sites
*wR*(*F*^2^) = 0.124	H atoms treated by a mixture of independent and constrained refinement
*S* = 1.01	*w* = 1/[σ^2^(*F*_o_^2^) + (0.0609*P*)^2^ + 0.6328*P*] where *P* = (*F*_o_^2^ + 2*F*_c_^2^)/3
2463 reflections	(Δ/σ)_max_ < 0.001
198 parameters	Δρ_max_ = 0.27 e Å^−^^3^
2 restraints	Δρ_min_ = −0.35 e Å^−^^3^

### Special details

**Table:**

Geometry. All e.s.d.'s (except the e.s.d. in the dihedral angle between two l.s. planes) are estimated using the full covariance matrix. The cell e.s.d.'s are taken into account individually in the estimation of e.s.d.'s in distances, angles and torsion angles; correlations between e.s.d.'s in cell parameters are only used when they are defined by crystal symmetry. An approximate (isotropic) treatment of cell e.s.d.'s is used for estimating e.s.d.'s involving l.s. planes.
Refinement. Refinement of *F*^2^ against ALL reflections. The weighted *R*-factor *wR* and goodness of fit *S* are based on *F*^2^, conventional *R*-factors *R* are based on *F*, with *F* set to zero for negative *F*^2^. The threshold expression of *F*^2^ > σ(*F*^2^) is used only for calculating *R*-factors(gt) *etc*. and is not relevant to the choice of reflections for refinement. *R*-factors based on *F*^2^ are statistically about twice as large as those based on *F*, and *R*- factors based on ALL data will be even larger.

### Fractional atomic coordinates and isotropic or equivalent isotropic displacement parameters (Å^2^)

**Table:**

	*x*	*y*	*z*	*U*_iso_*/*U*_eq_	
O1	0.06313 (12)	0.5135 (2)	−0.10419 (10)	0.0276 (4)	
O2	−0.00179 (13)	0.7915 (2)	−0.09219 (11)	0.0351 (4)	
O3	0.71122 (11)	0.6353 (2)	0.53463 (9)	0.0237 (4)	
O4	0.61240 (12)	0.7629 (2)	0.62568 (10)	0.0280 (4)	
N1	0.22094 (16)	1.0226 (3)	0.21975 (14)	0.0321 (5)	
H1NB	0.1839 (17)	1.109 (2)	0.1855 (15)	0.038*	
H1NA	0.2785 (12)	1.051 (3)	0.2629 (13)	0.038*	
C1	−0.00925 (17)	0.4946 (3)	−0.19589 (14)	0.0277 (5)	
H1C	−0.0122	0.3668	−0.2157	0.042*	
H1B	0.0153	0.5695	−0.2428	0.042*	
H1A	−0.0801	0.5349	−0.1915	0.042*	
C2	0.06020 (17)	0.6717 (3)	−0.05967 (15)	0.0215 (5)	
C3	0.14017 (16)	0.6815 (3)	0.03194 (14)	0.0201 (5)	
C4	0.14580 (16)	0.8404 (3)	0.08492 (14)	0.0205 (5)	
H4A	0.0976	0.9367	0.0624	0.025*	
C5	0.22104 (16)	0.8614 (3)	0.17076 (14)	0.0190 (5)	
C6	0.29167 (16)	0.7167 (3)	0.20412 (14)	0.0181 (5)	
C7	0.28276 (16)	0.5577 (3)	0.14953 (14)	0.0206 (5)	
H7A	0.3294	0.4592	0.1719	0.025*	
C8	0.20895 (17)	0.5381 (3)	0.06445 (14)	0.0212 (5)	
H8A	0.2052	0.4286	0.0287	0.025*	
C9	0.37584 (16)	0.7249 (3)	0.29319 (14)	0.0188 (5)	
C10	0.35757 (16)	0.7869 (3)	0.37973 (14)	0.0198 (5)	
H10A	0.2899	0.8339	0.3819	0.024*	
C11	0.43708 (17)	0.7805 (3)	0.46212 (14)	0.0209 (5)	
H11A	0.4235	0.8229	0.5204	0.025*	
C12	0.53657 (16)	0.7127 (3)	0.46029 (14)	0.0188 (5)	
C13	0.55552 (17)	0.6511 (3)	0.37448 (14)	0.0211 (5)	
H13A	0.6233	0.6040	0.3725	0.025*	
C14	0.47657 (16)	0.6582 (3)	0.29247 (14)	0.0215 (5)	
H14A	0.4909	0.6170	0.2342	0.026*	
C15	0.62115 (16)	0.7076 (3)	0.54930 (15)	0.0198 (5)	
C16	0.79774 (17)	0.6225 (3)	0.61760 (15)	0.0258 (5)	
H16A	0.8590	0.5660	0.5999	0.039*	
H16B	0.7760	0.5480	0.6662	0.039*	
H16C	0.8170	0.7446	0.6431	0.039*	

### Atomic displacement parameters (Å^2^)

**Table:**

	*U*^11^	*U*^22^	*U*^33^	*U*^12^	*U*^13^	*U*^23^
O1	0.0266 (9)	0.0302 (9)	0.0209 (8)	0.0058 (7)	−0.0052 (7)	−0.0085 (7)
O2	0.0427 (10)	0.0261 (9)	0.0284 (9)	0.0092 (8)	−0.0087 (8)	−0.0008 (7)
O3	0.0201 (8)	0.0284 (8)	0.0188 (8)	0.0022 (7)	−0.0030 (6)	−0.0014 (6)
O4	0.0283 (9)	0.0359 (9)	0.0183 (8)	0.0000 (7)	0.0019 (7)	−0.0053 (7)
N1	0.0321 (12)	0.0302 (11)	0.0304 (12)	0.0010 (9)	−0.0001 (9)	−0.0020 (9)
C1	0.0267 (12)	0.0343 (13)	0.0168 (11)	0.0013 (10)	−0.0059 (9)	−0.0066 (9)
C2	0.0238 (11)	0.0199 (11)	0.0203 (11)	0.0008 (9)	0.0038 (9)	0.0003 (9)
C3	0.0204 (11)	0.0224 (11)	0.0171 (11)	−0.0025 (9)	0.0033 (9)	−0.0003 (9)
C4	0.0203 (11)	0.0193 (10)	0.0213 (11)	0.0015 (9)	0.0035 (9)	0.0018 (9)
C5	0.0204 (11)	0.0201 (11)	0.0175 (10)	−0.0030 (9)	0.0062 (9)	−0.0013 (9)
C6	0.0166 (10)	0.0229 (11)	0.0155 (10)	−0.0017 (9)	0.0052 (9)	0.0000 (8)
C7	0.0196 (11)	0.0210 (11)	0.0205 (11)	0.0022 (9)	0.0032 (9)	0.0002 (9)
C8	0.0234 (11)	0.0201 (11)	0.0197 (11)	0.0001 (9)	0.0037 (9)	−0.0026 (9)
C9	0.0209 (11)	0.0168 (10)	0.0176 (10)	−0.0035 (9)	0.0019 (9)	−0.0006 (8)
C10	0.0191 (11)	0.0207 (11)	0.0191 (11)	−0.0001 (9)	0.0036 (9)	−0.0021 (9)
C11	0.0251 (12)	0.0202 (11)	0.0177 (11)	0.0001 (9)	0.0055 (9)	−0.0028 (8)
C12	0.0205 (11)	0.0163 (10)	0.0181 (11)	−0.0032 (9)	0.0014 (9)	0.0014 (8)
C13	0.0187 (11)	0.0243 (12)	0.0202 (11)	0.0001 (9)	0.0044 (9)	−0.0008 (9)
C14	0.0218 (11)	0.0274 (12)	0.0162 (10)	−0.0021 (9)	0.0060 (9)	−0.0048 (9)
C15	0.0227 (12)	0.0151 (10)	0.0204 (11)	−0.0042 (9)	0.0023 (9)	0.0021 (9)
C16	0.0220 (12)	0.0291 (12)	0.0216 (11)	0.0012 (10)	−0.0047 (9)	0.0014 (10)

### Geometric parameters (Å, º)

**Table:**

O1—C2	1.332 (2)	C6—C9	1.483 (3)
O1—C1	1.442 (2)	C7—C8	1.379 (3)
O2—C2	1.211 (2)	C7—H7A	0.9500
O3—C15	1.342 (2)	C8—H8A	0.9500
O3—C16	1.443 (2)	C9—C14	1.396 (3)
O4—C15	1.204 (2)	C9—C10	1.399 (3)
N1—C5	1.379 (3)	C10—C11	1.383 (3)
N1—H1NB	0.877 (5)	C10—H10A	0.9500
N1—H1NA	0.879 (5)	C11—C12	1.388 (3)
C1—H1C	0.9800	C11—H11A	0.9500
C1—H1B	0.9800	C12—C13	1.391 (3)
C1—H1A	0.9800	C12—C15	1.485 (3)
C2—C3	1.482 (3)	C13—C14	1.376 (3)
C3—C4	1.388 (3)	C13—H13A	0.9500
C3—C8	1.390 (3)	C14—H14A	0.9500
C4—C5	1.397 (3)	C16—H16A	0.9800
C4—H4A	0.9500	C16—H16B	0.9800
C5—C6	1.413 (3)	C16—H16C	0.9800
C6—C7	1.399 (3)		
			
C2—O1—C1	116.20 (16)	C7—C8—H8A	120.6
C15—O3—C16	115.67 (16)	C3—C8—H8A	120.6
C5—N1—H1NB	113.4 (17)	C14—C9—C10	118.07 (19)
C5—N1—H1NA	117.6 (17)	C14—C9—C6	118.81 (18)
H1NB—N1—H1NA	120 (2)	C10—C9—C6	123.01 (19)
O1—C1—H1C	109.5	C11—C10—C9	120.68 (19)
O1—C1—H1B	109.5	C11—C10—H10A	119.7
H1C—C1—H1B	109.5	C9—C10—H10A	119.7
O1—C1—H1A	109.5	C10—C11—C12	120.51 (18)
H1C—C1—H1A	109.5	C10—C11—H11A	119.7
H1B—C1—H1A	109.5	C12—C11—H11A	119.7
O2—C2—O1	122.46 (19)	C11—C12—C13	119.20 (19)
O2—C2—C3	125.17 (19)	C11—C12—C15	119.80 (18)
O1—C2—C3	112.37 (17)	C13—C12—C15	121.00 (19)
C4—C3—C8	120.21 (18)	C14—C13—C12	120.3 (2)
C4—C3—C2	117.99 (18)	C14—C13—H13A	119.9
C8—C3—C2	121.80 (18)	C12—C13—H13A	119.9
C3—C4—C5	121.22 (19)	C13—C14—C9	121.25 (18)
C3—C4—H4A	119.4	C13—C14—H14A	119.4
C5—C4—H4A	119.4	C9—C14—H14A	119.4
N1—C5—C4	117.78 (19)	O4—C15—O3	123.07 (19)
N1—C5—C6	123.24 (18)	O4—C15—C12	125.2 (2)
C4—C5—C6	118.97 (18)	O3—C15—C12	111.68 (17)
C7—C6—C5	118.25 (18)	O3—C16—H16A	109.5
C7—C6—C9	118.09 (18)	O3—C16—H16B	109.5
C5—C6—C9	123.65 (18)	H16A—C16—H16B	109.5
C8—C7—C6	122.60 (19)	O3—C16—H16C	109.5
C8—C7—H7A	118.7	H16A—C16—H16C	109.5
C6—C7—H7A	118.7	H16B—C16—H16C	109.5
C7—C8—C3	118.75 (19)		
			
C1—O1—C2—O2	2.4 (3)	C5—C6—C9—C14	−136.4 (2)
C1—O1—C2—C3	−177.63 (17)	C7—C6—C9—C10	−133.4 (2)
O2—C2—C3—C4	−0.5 (3)	C5—C6—C9—C10	47.5 (3)
O1—C2—C3—C4	179.46 (17)	C14—C9—C10—C11	−0.5 (3)
O2—C2—C3—C8	−179.8 (2)	C6—C9—C10—C11	175.68 (19)
O1—C2—C3—C8	0.2 (3)	C9—C10—C11—C12	0.1 (3)
C8—C3—C4—C5	1.0 (3)	C10—C11—C12—C13	0.0 (3)
C2—C3—C4—C5	−178.30 (18)	C10—C11—C12—C15	179.73 (18)
C3—C4—C5—N1	−179.38 (19)	C11—C12—C13—C14	0.3 (3)
C3—C4—C5—C6	−0.6 (3)	C15—C12—C13—C14	−179.44 (18)
N1—C5—C6—C7	178.43 (19)	C12—C13—C14—C9	−0.7 (3)
C4—C5—C6—C7	−0.2 (3)	C10—C9—C14—C13	0.8 (3)
N1—C5—C6—C9	−2.5 (3)	C6—C9—C14—C13	−175.55 (18)
C4—C5—C6—C9	178.85 (18)	C16—O3—C15—O4	1.7 (3)
C5—C6—C7—C8	0.8 (3)	C16—O3—C15—C12	−179.20 (16)
C9—C6—C7—C8	−178.35 (19)	C11—C12—C15—O4	−2.0 (3)
C6—C7—C8—C3	−0.5 (3)	C13—C12—C15—O4	177.7 (2)
C4—C3—C8—C7	−0.4 (3)	C11—C12—C15—O3	178.96 (18)
C2—C3—C8—C7	178.82 (19)	C13—C12—C15—O3	−1.3 (3)
C7—C6—C9—C14	42.7 (3)		

### Hydrogen-bond geometry (Å, º)

**Table:**

*D*—H···*A*	*D*—H	H···*A*	*D*···*A*	*D*—H···*A*
N1—H1*NB*···O2^i^	0.88 (1)	2.54 (2)	3.304 (3)	146 (2)
N1—H1*NA*···O4^ii^	0.88 (1)	2.33 (1)	3.147 (2)	155 (2)
C4—H4*A*···O2^i^	0.95	2.44	3.301 (3)	150

Symmetry codes: (i) −*x*, −*y*+2, −*z*; (ii) −*x*+1, −*y*+2, −*z*+1.
